# Next-Gen Restorative Materials to Revolutionise Smiles

**DOI:** 10.3390/bioengineering13020143

**Published:** 2026-01-27

**Authors:** John Yun Niu, Kelsey Xingyun Ge, Iris Xiaoxue Yin, Olivia Lili Zhang, Irene Shuping Zhao, Chun Hung Chu

**Affiliations:** 1 Faculty of Dentistry, The University of Hong Kong, Hong Kong SAR, China; niuyun@hku.hk (J.Y.N.); irisxyin@hku.hk (I.X.Y.); oliviazh@hku.hk (O.L.Z.); 2 School of Dentistry, Shenzhen University, Shenzhen 518055, China; kelseyge@szu.edu.cn

**Keywords:** nanomaterials, nanoparticles, bioactive, composite, caries, restoration

## Abstract

Recent breakthroughs in materials science have driven transformative advancements in restorative dentistry. Advanced dental materials, such as bioactive materials, nanocomposites, and fibre-reinforced composites, are attracting attention. Bioactive materials, such as calcium silicate-based cements and bioactive glass, represent a paradigm shift by interacting with biological tissues to stimulate regeneration. They promote hydroxyapatite formation, accelerating mineralisation in hard and soft tissues, and are pivotal tools in minimally invasive procedures due to their functions of structural support and biological interaction. Nanomaterials, especially nanocomposites with embedded nanoparticles, effectively address polymerisation shrinkage and wear in traditional composites. With just 1.5% shrinkage, a flexural strength over 150 MPa, and 44–60% higher wear resistance than conventional composites, they offer significant improvements. Nanocomposites also provide enamel-like translucency and a bond strength of 27–38 MPa to dentin, ensuring excellent aesthetics and durability—making them ideal for direct restorations. Fibre-reinforced composites with glass or polymer fibres balance aesthetics with strength and are increasingly used in restorations. Their high fracture resistance, which closely approaches that of a natural tooth, enables clinicians to preserve more healthy teeth during restoration, in line with the principles of modern conservative dentistry. Overall, bioactive materials enhance tissue repair, nanocomposites optimise form and function, and fibre-reinforced composites deliver strength without compromising aesthetics. As these materials transition from research to clinical practice, they promise longer-lasting treatments, fewer complications, and higher patient satisfaction. This narrative review aims to explore three types of advanced dental materials and their role in improving clinical outcomes.

## 1. Introduction

Restorative dentistry stands at the intersection of art and science, aiming to repair damaged teeth while balancing the functional durability, biological compatibility, and aesthetic appeal. For centuries, the field has relied on materials such as gold and silver amalgam to restore function and porcelain and composites for appearance. Traditional options are effective, but they can be improved. Silver amalgam has been common for more than a century due to its affordability and strength. However, while it is durable, its use is limited by aesthetic considerations and ongoing discussions regarding mercury-related safety concerns [1]. Resin-based composites emerged, offering tooth-coloured restorations. However, early composites struggled with wear, shrinkage, and bond failure, leading to secondary decay [2]. Porcelain, though aesthetically superior, requires aggressive tooth preparation and often fractures under stress [3]. These shortcomings highlight the need for materials that can balance strength, aesthetics, and biological support. Patients and clinicians demand natural-looking results, minimally invasive procedures, and materials that actively support oral health. Contemporary dental practice is undergoing a paradigm shift, driven by higher expectations from both patients and clinicians [2].

The progression from rudimentary fillings to advanced restorative systems reflects the evolving priorities of dentistry. Recent breakthroughs in materials science are redefining the boundaries of clinical practice, providing solutions that not only replicate the appearance of natural teeth but also interact dynamically with biological systems [4,5]. Notably, bioactive materials, nanocomposites, and fibre-reinforced composites are revolutionising restorative dentistry, establishing new benchmarks for clinical outcomes and patient satisfaction [5]. The advent of these innovative materials over the past two decades represents transformative solutions engineered to cooperate with the body, rather than to simply exist within it. These advancements address the priorities of contemporary dental care: minimally invasive procedures, long-term functional performance, and natural aesthetics [6].

Bioactive materials represent a significant step in restorative dentistry, transitioning from passive fillers to active participants in oral health [7]. By releasing ions such as calcium, phosphate, and fluoride, these materials stimulate the natural remineralisation of teeth and actively interact with biological tissues to promote regeneration. Notable examples, including calcium silicate-based cements and bioactive glass, facilitate hydroxyapatite formation and accelerate mineralisation in both hard and soft tissues [5]. Owing to their dual function as structural supports and bioactive agents, bioactive materials are indispensable in minimally invasive dentistry, where preservation of the natural tooth structure is paramount [6,8].

Equally transformative are nanomaterials, especially nanocomposites with embedded nanoparticles. Nanotechnology has enabled higher control over the properties of dental materials [9,10]. Nanocomposites, which incorporate nanoparticles into their matrix, effectively address the limitations of traditional composites, such as polymerisation shrinkage and wear resistance, by filling microscopic voids [11]. Their superior bond strength minimises microleakage, which is the primary cause of secondary decay [12]. Additionally, their exceptionally smooth surface texture resists plaque accumulation [13]. Hence, nanocomposites offer superior bond strength, aesthetics, and stain resistance, establishing them as the material of choice for direct restorative procedures [10].

Fibre-reinforced composites, incorporating glass or polymer fibres within a resin matrix, offer a balance of aesthetics and mechanical strength [14]. These materials provide a compelling alternative to traditional metal alloys, producing lightweight and durable restorations [15]. The flexibility and high fracture resistance of fibre-reinforced composites permit conservative tooth preparation, thereby preserving sound tooth tissue in accordance with the principles of modern conservative dentistry [16]. Additionally, their aesthetic adaptability facilitates seamless integration with natural dentition, effectively meeting the demands for visually pleasing outcomes in anterior and other highly visible regions [17]. Fibre-reinforced composites are increasingly utilised in restorations due to these combined advantages [18].

The integration of bioactive materials, nanocomposites, and fibre-reinforced composites represents a significant turning point in restorative dentistry. These advanced materials overcome traditional trade-offs, such as durability versus aesthetics and biocompatibility versus functionality. Ongoing research continues to refine their properties and broaden their clinical applications, with the potential to reduce complications such as secondary decay and postoperative sensitivity, while extending the longevity of restorations. For patients, these innovations translate into fewer dental appointments, reduced long-term costs, and confidence in aesthetics that both look and feel natural.

To provide clarity and direction, this review explicitly aims to (1) evaluate the properties, clinical performance, and mechanisms of bioactive materials, nanocomposites, and fibre-reinforced composites in restorative dentistry; (2) compare their advantages and limitations relative to traditional materials; and (3) discuss emerging trends and future perspectives in material development. The scope encompasses both scientific advances and practical clinical implications, offering a comprehensive overview for researchers, clinicians, and students interested in the evolving landscape of restorative dental materials.

## 2. Methods

This narrative review was conducted according to a structured approach to identify and synthesise the relevant literature on advanced restorative dental materials [19]. A comprehensive literature search was performed across multiple electronic databases, including PubMed, Scopus, and Web of Science, to ensure broad coverage and the identification of pertinent studies. The search strategy included all of the available literature up to 31 December 2025. Searching multiple databases was essential to minimise the risk of missing relevant studies. Relevant keywords were identified from the initial articles and were used to refine the search strategy. Commonly used keywords included “nanomaterials”, “bioactive”, “composite”, “restoration”, and “fibre-reinforced composites”. As this is a narrative review, the articles were selected based on their relevance and contribution to the synthesis rather than strict inclusion criteria. Based on the summary and synthesis of key findings from the selected articles, the evidence was integrated into the review to provide a comprehensive overview of the properties, clinical performance, and emerging trends in advanced restorative dental materials. Narrative reviews are designed to provide a comprehensive and integrated synthesis of the literature, rather than presenting data in a systematic or segmented manner. As such, the results and discussion are interwoven throughout the text to offer context, critical analysis, and interpretation, alongside the summary of the findings. This approach allows for a more cohesive and readable overview of current knowledge and emerging trends in the field. Relevant studies are cited as appropriate throughout the manuscript. No formal documentation of the literature search process was required for this narrative review; references are provided for all the included studies.

## 3. Bioactive Materials Introduce a New Era in Regenerative Dentistry

Bioactive materials have emerged as a cornerstone of modern restorative dentistry, bridging the gap between structural repair and biological regeneration [7,20]. In contrast to traditional inert materials that merely occupy space within a cavity or root canal, bioactive compounds interact with the oral environment to promote healing, remineralisation, and long-term tissue integration [21]. This section explores the science, clinical applications, and impact of these materials, with a focus on bioactive glass and calcium silicate-based cement, as well as their transformative role in redefining dental care.

The science of bioactivity represents a paradigm shift in restorative dentistry, transitioning from the use of passive fillers to dynamic healers [7]. Bioactive materials are defined by their ability to elicit specific biological responses at the interface between the material and host tissue, often through ion exchange, pH modulation, and the formation of hydroxyapatite (the mineral matrix of natural dentine and bone) [22]. Among these materials, bioactive glass and calcium silicate-based cement are predominant [23]. Bioactive glass contains silica, sodium, calcium, and phosphorus oxides. When exposed to bodily fluids, it undergoes controlled dissolution, releasing calcium and phosphate ions that react with the surrounding environment to form a hydroxycarbonate apatite layer. This layer chemically bonds to both hard and soft tissues, mimicking natural remineralisation [24]. Modern formulations such as NovaMin^®^ and BioMin^®^F incorporate fluoride to enhance the anticariogenic effects [25]. Calcium silicate-based cements, such as Biodentine™ and mineral trioxide aggregate, release calcium hydroxide upon setting, creating an alkaline environment (pH 12). This elevated pH neutralises bacterial acids and stimulates odontoblast differentiation (the cells responsible for dentin formation) [26,27].

The therapeutic power of bioactive materials is derived from their multifaceted mechanisms of action. Hydroxyapatite formation is a key process: bioactive glass releases ions that nucleate hydroxyapatite crystals, effectively sealing microgaps between the restoration and the tooth [24]. A study demonstrated that restorations incorporating bioactive glass reduced the bacterial microleakage by 61% compared to conventional resin composites [28]. Additionally, the high pH of calcium silicate cements creates an antimicrobial environment, significantly inhibiting the growth of pathogens such as *Streptococcus mutans* within 24 h [29]. Beyond antimicrobial effects, these materials promote tissue regeneration. Calcium silicate-based cements upregulate growth factors such as transforming growth factor-beta 1 (TGF-β1), which drive pulp stem cell differentiation into odontoblasts [30]. Clinical trials have reported a 92% success rate in preserving pulp vitality over two years when using Biodentine™ for direct pulp capping [31]. Furthermore, bioactive materials stimulate the formation of tertiary dentin, a reparative layer that shields the pulp from further injury [32]. This capability is invaluable in managing deep caries, where traditional materials might necessitate invasive root canal therapy.

Clinically, bioactive materials are revolutionising procedures ranging from endodontics to periodontal therapy. In vital pulp therapy, calcium silicate cements have become the gold standard for treating deep caries or traumatic pulp exposures [33]. Their ability to maintain the pulp vitality often eliminates the need for root canal treatment, preserving the tooth’s natural structure. In endodontics, bioactive sealers such as EndoSequence BC Sealer™ bond to dentin and continuously release calcium ions, reducing the reinfection risks [34]. Periodontal applications are equally promising: bioactive glass particles embedded in grafts (e.g., PerioGlas^®^) enhance osteoblast activity, accelerating bone regeneration in periodontal defects [35]. For minimally invasive restorations, bioactive liners placed beneath composites in deep cavities neutralise acids and promote dentin remineralisation [36], significantly improving the restoration longevity. Table 1 summarises the advantages of bioactive materials over conventional restorative materials such as silver amalgam and resin composites.

There are many advantages of bioactive materials over traditional options. Unlike inert materials such as amalgam or resin, bioactive compounds actively interact with tissues, facilitating the formation of hydroxyapatite at the material–tooth interface and reducing microleakage [24]. They also possess intrinsic antimicrobial properties, due to their pH-modulating effects [29], which conventional materials lack, unless supplemented with additives such as fluoride [20]. Clinically, this leads to fewer cases of postoperative sensitivity and inflammation. Moreover, bioactive materials are well aligned with the principles of minimally invasive dentistry, as their regenerative capabilities often minimise the need for extensive tooth preparation [38].

Despite their transformative potential, several challenges remain. Handling properties can be a significant limitation: some calcium silicate cements, such as mineral trioxide aggregate, require 4 h to set, complicating the clinical workflows [39]. Cost is another barrier, with bioactive formulations often costing two to three times more than conventional materials. Long-term data on their performance are also limited, as most studies focus on 2–5-year outcomes rather than decade-long durability [5]. However, ongoing innovations are addressing these limitations. Light-curable calcium silicate-based cements, such as TheraCal LC^®^, set fast in 20 s under curing light, streamlining procedures [40]. Researchers are also enhancing bioactive glass by incorporating nanoparticles to improve the mechanical strength without sacrificing the bioactivity [41]. Additionally, emerging technologies such as three-dimensional printed bioactive scaffolds may offer patient-specific solutions for large bone defects, utilising CAD/CAM precision to optimise the regenerative outcomes [42].

It is important to acknowledge several limitations in the current literature. Most studies on bioactive materials remain at the in vitro stage, and there is a risk of publication bias skewing the available evidence towards positive outcomes. Thus, further clinical trials with standardised methodologies are necessary to provide more robust and clinically relevant evidence for the adoption of bioactive materials in routine practice. Consequently, it remains difficult at present to draw definitive conclusions or make firm recommendations to clinicians regarding the routine use of bioactive materials in frontline clinical settings.

In summary, bioactive materials have redefined restorative dentistry, shifting the focus from repair to regeneration. By addressing biological, mechanical, and aesthetic demands, they epitomise the principles of minimally invasive and patient-centred care. As research advances, these materials are poised to form the foundation of “smart” restorations capable of adapting to oral environments, monitoring microbial activity, and even self-repairing [20]. This evolution underscores the broader trend in dentistry: the integration of materials science and biology to create solutions that not only restore aesthetics but also enhance the body’s innate healing capacity. Together with nanocomposites and fibre-reinforced composites (the topics of subsequent sections), bioactive materials are driving a revolution in which dental interventions are increasingly regenerative as well as restorative, ultimately promoting healthier and more durable outcomes for patients globally.

## 4. Nanocomposites for Aesthetic and Durable Restoration

Nanocomposites represent a significant progress in dental materials science, addressing the longstanding limitations of conventional resin-based composites while elevating the standards of aesthetic and functional restoration. By integrating nanoparticles (typically measuring between 1 and 100 nm) into resin matrices, these materials achieve a balance of strength, durability, and natural appearance that closely resembles the tooth structure [43]. This section explores the composition, mechanisms, and clinical implications of nanocomposites, underscoring their role as a cornerstone of modern restorative dentistry.

The evolution of nanocomposites stems from the inherent drawbacks of traditional composites, which have been used since the 1960s [44]. Early composites relied on larger filler particles (10–50 µm) suspended in a resin matrix, resulting in uneven wear, visible surface roughness, and polymerisation shrinkage [45]. Polymerisation shrinkage is a phenomenon where the material contracts by 2–5% during curing, creating microgaps that harbour bacteria. Nanocomposites overcome these shortcomings through their uniquely engineered nanostructure. By incorporating nanoparticles (nanoclusters) of silica, zirconia, or titania, these materials achieve filler loads of 70–80% by weight [46,47]. The high filler content minimises resin-rich areas, lowers polymerisation shrinkage to around 1.5%, and improves the load distribution, rendering restorations less likely to fracture [47]. For example, Filtek™ Supreme Ultra, a leading nanocomposite, contains zirconia–silica nanoclusters that bond closely with the resin, providing a flexural strength around 150 MPa [48], close to natural dentine 212.9 ± 41.9 MPa [49].

Mechanically, nanocomposites excel in both anterior and posterior applications. Table 2 compares the physical and mechanical properties of conventional composites with nanocomposites. Their wear resistance (a critical factor for molars) is 44–60% higher than that of traditional composites [50,51]. The bond strength to the tooth structure is also high; nanocomposites form hybrid layers with dentin through micromechanical interlocking and chemical bonding via adhesives, achieving bond strengths of 27–38 MPa [52]. This robust interface minimises microleakage, reducing secondary caries risk. For example, a big data analysis comparing nanocomposites to amalgam in restorations reported a 30% lower failure rate between 2014 and 2021 [53].

Aesthetic excellence is another hallmark of nanocomposites. Natural teeth derive their visual appeal from the complex interplay of light with enamel’s crystalline structure [56]. Conventional composites, which contain larger filler particles, tend to scatter light irregularly, resulting in restorations that appear either overly opaque or unnaturally glossy [55]. In contrast, nanoparticles approximate the size of hydroxyapatite crystals in enamel, allowing nanocomposites to replicate this light-reflecting behaviour. This nanoscale precision facilitates seamless colour matching and depth of translucency, critical for anterior restorations. Additionally, their smooth nano-textured surfaces resist staining from coffee, tea, and wine, a common issue with conventional composites [57,58].

The clinical versatility of nanocomposites is reshaping restorative protocols. In anterior applications, their aesthetic fidelity makes them ideal for diastema closures and veneers, where undetectable repairs are paramount. In posterior regions, their wear resistance supports the “bioemulation” approach, mimicking natural tooth biomechanics to prevent opposing tooth wear [59]. Flowable nanocomposites, with reduced viscosity, are increasingly used for minimally invasive fissure sealants and cervical abrasion lesions, as their ability to penetrate submicron tooth irregularities surpasses that of conventional materials [60,61]. A 2019 case series documented a 70% retention rate for flowable nanocomposite sealants after 1 year, outperforming resin-modified glass ionomers [62]. Despite these advances, challenges persist. The high surface energy of nanoparticles can lead to agglomeration during manufacturing, compromising their homogeneity [63]. Clinicians also face a learning curve in handling nanocomposites, as their thick consistency requires precise layering techniques to avoid voids [64]. However, manufacturers address these issues through pre-heating systems that improve the flow and workability [65]. Cost remains a concern, with nanocomposites priced higher than traditional options, although their longevity may offset the initial expenses over time.

The next generation of nanocomposites integrates bioactive and smart functionalities. Calcium phosphate nanoparticles are being added to promote remineralisation, blurring the line between restorative and preventive care [66]. Antimicrobial nanocomposites embedded with silver or zinc oxide nanoparticles show promise in reducing recurrent caries, with in vitro studies demonstrating a more than 95% reduction in *Streptococcus mutans* biofilm formation [67]. Self-healing nanocomposites, although experimental, utilise microcapsules of resin monomer that rupture under stress, autonomously repairing microcracks, a potentially significant improvement for restoration longevity [68].

Although nanocomposite materials are now widely used in clinical practice, their history is shorter than that of traditional amalgam or conventional resin materials [44]. Therefore, longer-term observation is required to confirm their sustainability and efficacy. It is noteworthy that, in cases where aesthetics are a primary concern, clinicians already choose nanocomposite materials [69]. However, for posterior teeth that must withstand significant occlusal forces, there remains debate regarding the use of nanocomposites versus traditional amalgam [70]. Furthermore, the adoption of new bulk-fill composite materials will require long-term clinical studies to fully establish their reliability and effectiveness.

In summary, nanocomposites have redefined the possibilities of direct restorative dentistry. By marrying nanotechnology, they address the tripartite demands of strength, aesthetics, and durability that were once considered mutually exclusive [43]. As research progresses toward bioactive and adaptive formulations, nanocomposites are poised to become not just a material choice but a comprehensive solution for preserving natural dentition in an era where patients increasingly refuse to compromise between health and beauty. Their integration with the other pillars of next-generation materials, bioactive agents, and fibre-reinforced composites, heralds a future where dental restorations are indistinguishable from nature’s design in both form and function.

## 5. Fibre-Reinforced Composites with Enhanced Strength and Aesthetics

Fibre-reinforced composites have emerged as a transformative solution in restorative dentistry, reconciling the historical divide between high-strength prosthetics and natural aesthetics. By embedding continuous fibres or discontinuous fibre networks within a resin matrix, these materials achieve flexural strengths comparable to metal alloys while maintaining the translucent tooth-like appearance of composites [17]. This section examines the composition, clinical applications, and paradigm-shifting benefits of fibre-reinforced composites, positioning them as indispensable tools for conservative patient-centred care.

The fundamental innovation of fibre-reinforced composites lies in their hybrid structure, which combines the stress-bearing capacity of fibres with the mouldable aesthetics of resin. Most fibre-reinforced composites use glass fibres or ultra-high-molecular-weight polyethene fibres, which are pre-impregnated with dimethacrylate resin [15]. When polymerised, the fibres act as a reinforcing skeleton, redistributing occlusal forces across the restoration. Unlike bulk metals, fibre-reinforced composites exhibit anisotropic flexibility, allowing them to absorb and release energy during chewing, which reduces the risk of root fractures in abutment teeth [71]. This unique property stems from the fibres’ alignment: unidirectional fibres provide maximum strength along the restoration’s long axis, while woven mats offer multidirectional reinforcement for complex geometries such as multi-unit bridges [72].

Aesthetically, fibre-reinforced composites resolve the “opacity dilemma” of metal–ceramic crowns. Traditional metal frameworks require opaque masking layers to hide their metallic shine, resulting in restorations that appear flat and artificial under natural light. However, fibre-reinforced composites leverage the translucency of their resin matrix and the light-conducting properties of glass fibres to mimic the enamel’s depth and vitality [17]. The fibres themselves can be tinted to match the dentin shades, enabling restorations to blend seamlessly with the adjacent teeth. Furthermore, fibre-reinforced composites avoid the grey gumline discolouration associated with porcelain-fused-to-metal restorations, making them particularly advantageous for anterior applications. Table 3 summarises the advantages of fibre-reinforced composites over conventional materials.

Fibre-reinforced composites are revolutionising indirect restorations through minimally invasive protocols. Fibre-reinforced composites inlays and onlays preserve a sound tooth structure by bonding directly to the remaining enamel and dentin [85]. This approach aligns with the bioemulation philosophy, using adhesive techniques to restore, rather than replace, natural tooth biomechanics [74]. In fixed prosthodontics, fibre-reinforced composite bridges offer a metal-free alternative for replacing 1–2 missing teeth [76]. The applications extend beyond conventional restorations. Periodontists employ fibre-reinforced composite splints to stabilise the mobile teeth, using materials such as everStick^®^ Perio, which bonds to both the enamel and composite [77]. Orthodontists utilise fibre-reinforced composites for fixed retainers, offering enhanced comfort and superior aesthetics compared to traditional stainless-steel wires [78]. Even in implantology, fibre-reinforced composites are gaining traction, as customisable abutments avoid the shining-through effect of metal abutments using an all-ceramic or composite suprastructure [79].

The shift toward fibre-reinforced composites is driven by their technical versatility. Chairside systems enable same-day restorations through CAD/CAM milling of short fibre-reinforced composite blocks, combining the speed of digital dentistry with the material’s inherent benefits [84]. For lab-fabricated prostheses, heat-pressing techniques allow fibres to be oriented precisely along the stress trajectories, optimising the mechanical properties [85]. This adaptability has made fibre-reinforced composites particularly valuable in complex cases, such as patients with parafunctional habits (e.g., bruxism), where their energy-absorbing properties protect both the restoration and opposing dentition.

Despite these advantages, fibre-reinforced composites present unique challenges. Delamination—the separation of fibres from the resin matrix—can occur under prolonged moisture exposure [86], although modern silane coupling agents can solve this problem [87]. Cost remains a consideration, with fibre-reinforced composites restorations priced higher than porcelain-fused metal equivalents, but their minimally invasive nature often reduces the long-term expenses by preserving the tooth structure and preventing future complications.

The future of fibre-reinforced composites lies in their multifunctional and bioactive innovations; three-dimensional printing technologies now enable the deposition of continuous fibres in custom patterns, opening possibilities for patient-specific reinforcing structure optimisation [88].

Whether fibre-reinforced composites can fully replace traditional metal or ceramic materials in fixed dental prostheses, as well as in root canal posts and removable dentures, remains an important question [89]. More long-term clinical data and systematic analysis are needed. In summary, fibre-reinforced composites epitomise the evolution of restorative dentistry from a discipline of replacement to one of preservation and biomimicry. By uniting the mechanical prowess of engineering materials with the subtleties of natural aesthetics, they fulfil the modern patient’s demand for durable invisible restorations. As part of the triad of next-generation materials, alongside bioactive agents and nanocomposites, fibre-reinforced composites are redefining clinical success not merely as survival rates but as the preservation of vitality, function, and confidence. Their continued refinement will further erase the lines between artificial and natural, ensuring that tomorrow’s smiles are not only restored but also revitalised.

## 6. Advanced Dental Materials with Antimicrobial Properties

Oral diseases, such as caries and periodontitis, are fundamentally infectious in nature [90]. Therefore, the ability of advanced restorative materials to actively inhibit microbial colonisation and biofilm formation represents a pivotal advancement in dental care. By presenting the antimicrobial properties in a separate section, we aim to underscore the paradigm shift from passive restoration to active infection control, highlighting that antimicrobial functionality is now a core requirement—rather than merely an added benefit—in the contemporary management and prevention of oral diseases. The integration of antimicrobial functionality into restorative materials has become a critical frontier in combating secondary caries and peri-restorative infections [91]. Bioactive materials, nanocomposites, and fibre-reinforced composites each utilise distinct mechanisms to inhibit microbial proliferation, thereby complementing their structural functions and contributing to improved long-term clinical outcomes.

Bioactive materials leverage ion release to create hostile environments for pathogens. Calcium silicate cements such as Biodentine™ elevate the pH to 12 via calcium hydroxide release, neutralising acidogenic bacteria such as *Streptococcus mutans* [29]. Bioactive glass releases sodium ions that disrupt bacterial membranes while fostering hydroxyapatite deposition, sealing microgaps that harbour biofilms [92].

Nanocomposites incorporate antimicrobial nanoparticles to achieve targeted antibacterial effects. Silver nanoparticles in products such as Filtek™ Supreme disrupt bacterial cell walls and DNA replication [93]. Zinc oxide nanoparticles quench oxidative stress in microbial cells, while quaternary ammonium monomers electrostatically rupture biofilms [93,94].

Fibre-reinforced composites achieve antimicrobial effects through surface modifications. Silanised glass fibres in fibre-reinforced composites can be loaded with chlorhexidine, which gradually releases to inhibit plaque accumulation [95].

Overall, these materials address microbial challenges across clinical scenarios—bioactive agents in deep caries, nanocomposites in high-risk margins, and fibre-reinforced composites in prostheses that are prone to biofilm accumulation. By reducing reliance on adjunctive antimicrobials, they lower the antibiotic resistance risks while extending the restoration lifespans, particularly benefiting elderly, diabetic, or xerostomia patients. This triad of antimicrobial strategies marks a paradigm shift from passive restoration to active infection control in dental care.

## 7. Conclusions

In conclusion, the landscape of restorative dentistry is rapidly transforming through the introduction of advanced dental materials. This review highlights that bioactive materials show promise in promoting tissue repair and may contribute to improving pulp vitality and secondary caries prevention. Nanocomposites demonstrate enhanced handling properties and superior aesthetic outcomes, although their long-term durability requires further clinical validation. Fibre-reinforced composites offer a viable alternative to traditional materials in certain applications, combining favourable aesthetics with increased fracture resistance.

Clinicians should be aware that most of the current evidence is derived from in vitro studies or short-term clinical trials, and the heterogeneity in study designs complicates the direct comparison of outcomes. For clinical practice, it is recommended that clinicians carefully select restorative materials based on the specific clinical scenario, patient needs, and the available evidence. Future research should prioritise large-scale standardised clinical trials to assess the long-term performance, safety, and cost-effectiveness of these novel materials across diverse patient populations. Development of clear evidence-based guidelines will be essential to support clinicians in integrating these innovations into everyday practice.

## Figures and Tables

**Table 1:** Advantages of bioactive materials over conventional materials.

Parameter	Bioactive Materials [Reference]	Conventional Materials [Reference]
Biological Impact	Promotes remineralisation and healing [21]	Inert; no interaction with tissues [20]
Marginal Seal	Forms hydroxyapatite bond, sealing gaps [21]	Prone to microleakage due to shrinkage [24]
Antimicrobial Effect	pH-mediated antimicrobial activity [21]	None (unless there are additives such as fluoride) [20]
Pulp Compatibility	Soothes pulp and reduces inflammation [21]	Risk of irritation and postoperative sensitivity [37]

**Table 2:** Physical and mechanical properties of conventional composites and nanocomposites.

Parameter	Nanocomposites [Reference]	Conventional Composites [Reference]
Filler Size	1–100 nm nanoparticles/nanoclusters [43]	10–50 µm macrofillers [54]
Polymerisation Shrinkage	1.5% (reduced microleakage) [47]	2–5% (higher risk of gaps) [45]
Flexural Strength	>150 MPa (matches dentin) [48]	80–100 MPa (conventional composites) [48]
Wear Resistance	>44–60% than conventional composite [50]	Prone to occlusal wear [51]
Translucency	Enamel-like translucency [55]	Opaque/glossy surface [55]
Bond Strength	27–38 MPa (hybrid layer with dentin) [52]	19–25 MPa (weaker interface) [52]

**Table 3:** Advantages of fibre-reinforced composites over conventional materials.

Parameter	Fibre-Reinforced Composites [Reference]	Conventional Materials [Reference]
Mechanical Strength	High flexural strength, anisotropic flexibility absorbs occlusal stress [14]	Metals: high rigidity but no flexibility Porcelain: brittle and prone to fracture [14]
Aesthetic Integration	Translucent fibres mimic enamel; colour-matched to dentin [17]	Metals: grey margins Porcelain: need to mask metal frameworks [73]
Tooth Preservation	Use of adhesive preserves healthy structure [74]	Full crowns require aggressive preparation [75]
Clinical Applications	Bridges, onlays, splints, and implant prostheses [74,76,77,78,79]	Metals: limited to crowns/bridges; Porcelain: mostly crowns/veneers [80]
Biocompatibility	Metal-free; reduced allergy risk [76]	Metals: may cause hypersensitivity [81] Porcelain: requires metal substrates [80]
Longevity	High fatigue resistance; good survival rate for bridges [72]	Metals: may cause opposing tooth wear [82] Porcelain: easy to fracture [83]
Additional Benefits	CAD/CAM customisation [84]	-

## Data Availability

The original contributions presented in the study are included in the article material; further inquiries can be directed to the corresponding authors.
